# Characterization of Force Distribution and Force Chain Topology in Asphalt Mixtures Using the Discrete Element Method

**DOI:** 10.3390/ma18102347

**Published:** 2025-05-18

**Authors:** Sudi Wang, Jianxia Wang, Jie Wang, Jian Xu, Yinghao Miao, Qing Ma, Linbing Wang, Tao Liu

**Affiliations:** 1 Research Institute of Highway Ministry of Transport, Beijing 100088, China; sd.wang@rioh.cn (S.W.);; 2 Jiangxi Transportation Investment Group Co., Ltd., Nanchang 330025, China; 3 National Center for Materials Service Safety, University of Science and Technology Beijing, Beijing 100083, China; 4 School of Environmental, Civil, Agricultural and Mechanical Engineering, University of Georgia, Athens, GA 30602, USA

**Keywords:** asphalt mixture, contact force, load bearing contribution, force chain, topology characteristics, discrete element method

## Abstract

The force chain network within asphalt mixtures serves as the primary load-bearing structure to resist external forces. The objective of this study is to quantitatively characterize the contact force distribution and force chain topology structure. The discrete element method (DEM) was employed to construct simulation models for two stone matrix asphalt (SMA) and two open-graded friction course (OGFC) mixtures. Load distribution characteristics, including average contact force, load bearing contribution and contact force angle, and force chain topological network parameters, clustering coefficient, edge betweenness and average path length, were analyzed to elucidate the load transfer mechanisms. The findings of the present study demonstrate that the average contact force between aggregate–aggregate contact types in specific particle sizes significantly exceeds the average contact force of the same particle size aggregates. For SMA16 and OGFC16 asphalt mixtures, the load-bearing contribution of aggregates initially increases and then decreases with decreasing particle size, peaking at 13.2 mm. SMA13 and OGFC13 mixtures demonstrate a consistent decline in load bearing contribution with decreasing aggregate size. The analysis of the force chain network topology of the asphalt mixture reveals that SMA mixtures exhibited higher average clustering coefficients in force chain topological features in comparison to OGFC mixtures. It indicates that SMA gradations have superior skeletal load-bearing structures. While the maximum nominal aggregate size minimally influences the average path length with a relative change rate of 3%, the gradation type exerts a more substantial impact, exhibiting a relative change rate of 7% to 9%. These findings confirm that SMA mixtures have more stable load-bearing structures than OGFC mixtures. The proposed topological parameters effectively capture structural distinctions in force chain networks, offering insights for optimizing gradation design and enhancing mechanical performance.

## 1. Introduction

The force chain network in asphalt mixtures represents the primary load-bearing structure that is formed in asphalt mixtures under the action of external loads, thereby exerting a substantial influence on the performance of asphalt mixtures [1]. The macroscopic mechanical response of asphalt mixtures can be considered a reflection of the meso-scale load transfer mechanism. The study of the load transfer network in the meso-scale structure of asphalt mixtures provides a novel perspective through which to examine the macroscopic mechanical properties of asphalt mixtures [2,3]. Consequently, effective characterization of the force chain network of asphalt mixtures is conducive to a more profound understanding of the mechanical behavior of asphalt mixtures from the meso-scale, which is of great significance for the optimal design of asphalt mixtures.

In order to explore the asphalt mixture bearing mechanism, the internal load distribution characteristics of asphalt mixtures were investigated. Liu et al. [4] utilized a discrete element method (DEM) to reconstruct asphalt mixtures, thereby developing a numerical simulation model of asphalt mixtures. The force chain distribution inside the aggregate, inside the mortar, and at the aggregate–mortar interface was analyzed. Chang et al. [5,6,7] employed the DEM to reconstruct digital specimens of asphalt mixtures, and quantitatively analyzed the evolution and distribution of force chains inside asphalt mixtures. The experimental findings demonstrate that the spatial distribution of contact forces within asphalt mixtures is characterized by anisotropy. Tan et al. [8] utilized digital image technology in conjunction with DEM to investigate and analyze the load transfer characteristics within the asphalt mixture skeleton structure. The research further explored and examined the influence of the skeleton structure on the contact force between aggregate and mortar. It was found that the load transfer in the asphalt mixture is predominantly facilitated by the primary skeleton aggregate particles. Wang et al. [2] employed reconstructed aggregate particles to generate discrete element specimens, utilizing this method to investigate the effect of elongated and flat particle content on the load transfer characteristics of asphalt mixtures during compaction. The simulation test results demonstrate that the overall load carrying capacity of the mixture becomes weaker with the increase of elongated and flat particle content. Wei et al. [9] characterized the force chain of asphalt mixtures by developing a three-dimensional finite element simulation model. The results obtained from this model indicated that the stress concentration region in the asphalt mortar matrix was regarded as the main load transfer region between the coarse aggregates. Shi et al. [10] developed a numerical model of asphalt mixtures with different gradations considering the real aggregate morphology and conducted uniaxial compression tests, which revealed that aggregates with 4.75–9.5 mm particle size have both load-bearing and filling functions, and their roles are significantly affected by the gradation. It was also found that the internal aggregate structure could be optimized and adjusted under stress. Yao et al. [11] employed discrete element method (DEM) simulations to analyze stress transmission mechanisms within asphalt mixtures, with a focus on quantitatively evaluating their splitting strength performance. It was found that in comparison with AC13 (asphalt concrete with the nominal particle size of 13.2 mm) gradation, AC20 (asphalt concrete with the nominal particle size of 20 mm) gradation asphalt mixture results in a greater proportion of coarse aggregates carrying the load.

Under the action of external loads, a contact network structure that transmits external loads was formed between particles in asphalt mixtures. This network structure is similar to the topology in complex networks and is called the contact force chain structure [12]. Many studies have been carried out to investigate the force chain structure in granular mixtures. Kruyt [13] initially proposed the definitions of strong contact and weak contact, which were based on the magnitude of the contact force and the magnitude of the force in the direction of contact. Voivret et al. [14] investigated the anisotropy of the force chain network within a granular system on this basis. On the basis of force chain structure research, some scholars have carried out relevant studies on the contact force distribution characteristics in the granular matter. Mueth et al. [15] provided a fitting function for the contact force distribution in mixed granular matter. Sun et al. [16] studied the contact force distribution characteristics within the granular matter and quantitatively analyzed the angle of the force chain structure. The results of the study found the directionality of the strong force chain structure, and on this basis, a strong force chain criterion for the granular system was proposed under uniaxial compression and biaxial compression loading by carrying out discrete element simulation experiments [17]. Feng et al. [18] investigated the effect of particle size dimensions on the mechanical and geometrical structural properties of mixed particle systems using DEM (Particle Flow Code 5.0). The results demonstrated that the number of contacts between particles varied with the degree of particle size dispersion, while the cumulative distribution of contact forces between particles remained unaffected by the degree of particle size dispersion. Danylenko et al. [19] conducted a study on the contact force characteristics of individual particles under external loading. The results revealed the relationship between the contact forces between particles of the mix during dynamic loading. Kondic et al. [20] investigated the evolution of the contact force network with loading time in mixes under different external loads. Yu et al. [12,21,22] investigated the topological features of the force chain network in aggregate blend utilizing discrete element method and through complex network theory. It was found that the average path length of SMA-16 is longer than that of AC-25, indicating that SMA-16 has better load transfer ability and skeleton properties. Liu et al. [23] evaluated the affinity between aggregate skeleton topology and rutting performance of asphalt mixture based on network theory. The findings of this research indicated that, in terms of the realistic affinity with rutting performance, the combined feature demonstrated the most optimal performance, exhibiting an average correlation of 0.55. Yang et al. [24] investigated the microscopic fracture characteristics of recycled asphalt mixtures through the force chain distribution using DEM. It was found that the emulsified asphalt mortar–aggregate interface was the critical weak position of the mixture fracture. Additionally, the failure of the tension chain was the main destructional form of the asphalt mixture fracture.

Asphalt mixtures have significant granularity, and therefore, the discrete element method (DEM) is considered to be an effective means to study discontinuities in the distribution of asphalt mixture components and discontinuities in the mechanical properties of the interfaces between different components [25]. Typically, researchers analyzed the meso-mechanical properties of asphalt mixtures using DEM. Based on DEM, Liu et al. [26] simulated the mechanical behavior of asphalt mixtures under low temperature conditions using the linear elasticity model to simulate coarse aggregates, and Burger’s model instead of the linear elasticity model to simulate asphalt mortar. Ma et al. [27,28,29] found that the better the embeddedness between aggregate particles, the better the high-temperature stability, through the DEM simulation of asphalt mixtures. Concurrently, related studies on cracking behavior and splitting strength of asphalt mixtures were carried out based on DEM [24,30,31,32]. Relevant researchers have also studied the structural characteristics of asphalt mixtures utilizing the DEM [33,34,35]. Chen et al. [36] simulated rotary compaction tests of mixes by simulating aggregates through particles of simple morphology. Gong et al. [37,38] explored the kinematic properties of aggregate particles during the compaction process with the help of the real morphology model of aggregate particles obtained via X-ray CT and optical scanning, which were imported into Particle Flow Code 5.0 software to carry out DEM tests to simulate the SGC test of asphalt mixtures. Chen et al. [39] investigated the mobility of loose-state asphalt mixtures in a slump cylinder using the DEM simulation. The findings indicated that the mobility of loose state asphalt mixtures was primarily determined by the combination of lubricating friction and bonding between particles. Liang et al. [3] analyzed the contact characteristics of the asphalt mixture particle contact network by reconstructing the asphalt mixture in three dimensions by computed tomography scanning. The contact number of stone matrix asphalt with a maximum nominal particle size of 16 mm was found to be significantly higher in comparison to that of asphalt concrete with a maximum nominal particle size of 16 mm. Wang et al. [40] investigated the force chain evolution and aggregate movement in asphalt mixtures using DEM. It was found that gradation variations in asphalt mixtures can alter the load-bearing contribution of aggregate particles. SMA gradations exhibit enhanced ability to withstand and transmit external loads compared to OGFC gradations.

**Figure 1:**
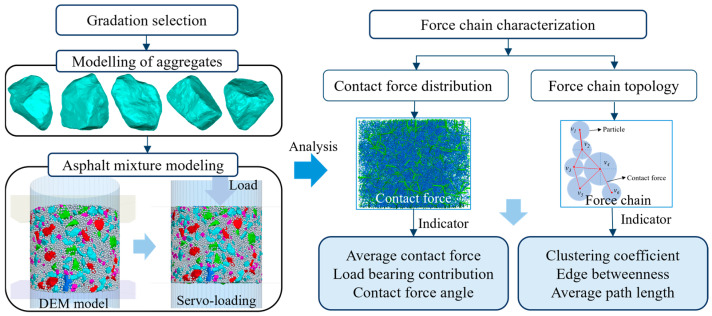
The methodology flowchart of this paper.

As demonstrated by the preceding studies, the DEM has emerged as a more suitable tool for the analysis of asphalt mixtures. While numerous scholars have conducted research on the load transfer mechanism of asphalt mixtures, their studies are constrained by the quantification and evaluation of the contact force magnitude. The research and analysis of the entire force chain structure and the force distribution characteristics remain in their initial stages and lack comprehensive depth. The topology in complex networks is an effective tool to characterize the structure of the force chain, and related studies have confirmed the feasibility of this method [22,23]. While previous studies have focused on contact force magnitudes, this work quantifies force chain topology (e.g., clustering coefficient, edge betweenness) to assess structural stability, which has not been comprehensively addressed. This paper focuses on the contact force distribution and the topological structure characteristics of the force chain network in asphalt mixture. The stone mastic asphalt (SMA) gradation provides excellent rutting resistance and durability due to its dense skeleton and high asphalt dosage, and is suitable for heavy traffic areas, while open graded friction course (OGFC) gradation is designed for skid resistance in rainy areas by achieving efficient drainage and noise reduction through its open graded pore structure. Considering the functional characteristics and performance advantages of SMA and OGFC, they are selected as experimental objects to investigate the load transfer mechanism of the dense skeleton and permeable structure [40]. Three-dimensional laser scanning is employed to obtain the real morphology of aggregate particles, with Particle Flow Code (PFC) 5.0 software then being used to generate a four-graded asphalt mixture discrete element numerical model. The load transfer response during the simulation experiment is tracked using the FISH function. The analysis of the internal load transfer mechanism of asphalt mixtures under external load is facilitated by the interaction force between particles and the contact force network. The topological structure of the contact force network is analyzed using the complex network theory, which gives the quantitative statistical indexes of force value and structure of the internal force chain network of asphalt mixtures. Figure 1 depicts the steps, from asphalt mixture model compaction to analysis of the simulation results.

## 2. Materials and Methods

### 2.1. Materials and Gradations

SMA and OGFC are two typical asphalt mixture types widely used in different pavement engineering scenarios, and 13.2 mm and 16 mm are the common nominal maximum particle sizes. Therefore, two stone mastic asphalt (SMA) gradations and two open graded friction course (OGFC) gradations were selected with nominal maximum aggregate grain sizes of 13.2 mm and 16 mm, respectively. The SMA graded asphalt mixture with a nominal maximum particle size of 16 mm is denoted SMA16, and the other three asphalt mixtures are represented in the same way. These four mixes are chosen to represent the different types of asphalt mixtures with different particle sizes and gradation types in actual projects, which facilitates the study of the effects of changes in particle size and gradation on the force chain characteristics and mechanical properties. According to Technical Specifications for Construction of Highway Asphalt Pavement (JTG F40-2004) [41], the Marshall design method was used to determine the grading curves and optimal asphalt–aggregate ratio of the four asphalt mixtures. The optimal asphalt-aggregate ratio and gradation of asphalt mixtures are shown in Table 1. In this study, the coarse aggregate is basalt, the fine material is sand, and the basic characteristics of the aggregate are shown in Table 2.

### 2.2. DEM Simulation of Asphalt Mixtures

#### 2.2.1. Contact Parameters of Simulation Model

(1) Linear contact model of coarse aggregates

**Figure 2:**
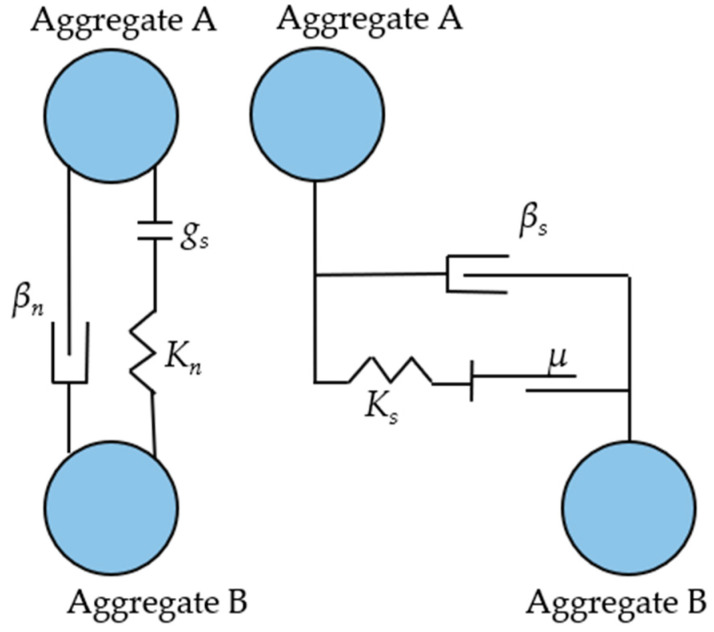
Linear contact model of aggregate. (Note: To distinguish particles, the two aggregate particles in contact with each other are named Aggregate A and Aggregate B).

Since the mechanical response of coarse aggregate is close to elasticity, a linear contact model is utilized to characterize the contact model between the coarse aggregate–aggregate [42,43], as illustrated in Figure 2. The coarse aggregate units are represented by clump units.

In Figure 2, $g_{s}$ is the gap between particles; $\beta_{s}$ is the tangential critical damping ratio; and $\beta_{n}$ is the normal critical damping ratio. $K_{s}$ and $K_{n}$ are tangential stiffness and normal stiffness of the tandem, respectively, which are calculated as in Equation (1).

(1) $$\left\{\begin{array}{l} K_{n}=\frac{k_{n}^{A}k_{n}^{B}}{k_{n}^{A}+k_{n}^{B}}\\ K_{s}=\frac{k_{s}^{A}k_{s}^{B}}{k_{s}^{A}+k_{s}^{B}} \end{array}\right.$$

where $K_{s}^{A}$ and $K_{n}^{A}$ are the normal stiffness and tangential stiffness of particle *A*; $K_{s}^{B}$ and $K_{n}^{B}$ are the tangential stiffness and normal stiffness of particle *B*, respectively.

Under axial force and shear loading, the tangential contact stiffness can be transformed by the normal contact stiffness, as demonstrated in Equation (2).

(2) $$k_{s}=\frac{k_{n}}{2(1+v)}$$

where v is the Poisson’s ratio. The relationship between the macroelastic model and normal stiffness, as demonstrated in Equation (3).

(3) $$k_{n}=\frac{AE_{c}}{L}$$

If the two particles have the same tangential and normal stiffness, the stiffness of particles *A* and *B* can be obtained according to Equations (1)–(3), as shown in Equation (4).

(4) $$\left\{\begin{array}{l} k_{n}^{A}=k_{n}^{B}=\frac{2AE_{c}}{L}\\ k_{s}^{A}=k_{s}^{B}=\frac{AE_{c}}{L(1+v)} \end{array}\right.$$

where $E_{c}$ is Young’s contact elasticity model; *R* is the radius of the contact unit; and $L=2\bar{R}=R^{A}+R^{B}$ is the equivalent length of the elastic beam.

The expressions for the moment of inertia I and cross-sectional area *A* of an elastic beam are given in Equation (5).

(5) $$\left\{\begin{array}{l} A=\left\{\begin{array}{l} {\left(2\bar{R}\right)}^{2}\;(3D)\\ 2\bar{R}t\;(2D) \end{array}\right.\\ I=\left\{\begin{array}{l} \frac{4}{3}{\bar{R}}^{4}\;(3D)\\ \frac{2}{3}{\bar{R}}^{3}t\;(2D) \end{array}\right. \end{array}\right.$$

The calculation of the meso-scale parameters in the linear contact stiffness model is undertaken according to Equation (6).

(6) $$\left\{\begin{array}{l} k_{n}^{A}=k_{n}^{B}=2E_{c}\left\{\begin{array}{l} L\;(3D)\\ t\;(2D) \end{array}\right.\\ k_{s}^{A}=k_{s}^{B}=\frac{E_{c}}{L(1+v)}\left\{\begin{array}{l} L\;(3D)\\ t\;(2D) \end{array}\right. \end{array}\right.$$

where $k_{s}^{A},k_{n}^{A},k_{s}^{B},$ and $k_{n}^{B}$ are interpreted as in Equation (1).

The relationship between the microscopic contact stiffness and macroscopic modulus of elasticity of the unit in the above equation, when combined with the macroscopic modulus of elasticity of the aggregate, can be used to obtain the stiffness parameters in the linear contact model. According to references [42,43], the Poisson’s ratio, shear modulus and modulus of elasticity of the coarse aggregate were determined to be 0.25, 22.2 GPa and 55.5 GPa, respectively. Additionally, the friction coefficient is 0.5.

(2) Burger’s model of asphalt mortar

**Figure 3:**
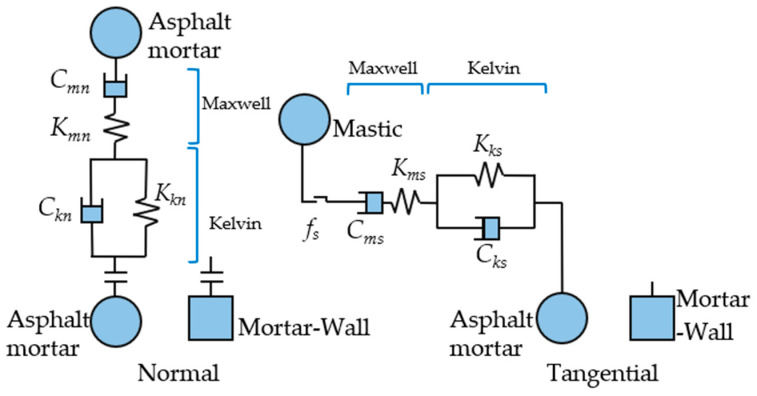
Burger’s contact model.

The asphalt mortar is viscoelastic and Burger’s contact model was chosen to characterize the contact model between asphalt mortar units [2,42,43], as shown in Figure 3. The relationship between the parameters in Burger’s model is expressed in Equation (7).

(7) $$\left\{\begin{array}{l} C_{mn}=\eta_{1}\left\{\begin{array}{l} t\;(2D)\\ L\;(3D) \end{array}\right.\;K_{mn}=E_{1}\left\{\begin{array}{l} t\;(2D)\\ L\;(3D) \end{array}\right.\\ C_{kn}=\eta_{2}\left\{\begin{array}{l} t\;(2D)\\ L\;(3D) \end{array}\right.\;K_{kn}=E_{2}\left\{\begin{array}{l} t\;(2D)\\ L\;(3D) \end{array}\right.\\ C_{ms}=\frac{\eta_{1}}{2(1+v)}\left\{\begin{array}{l} t\;(2D)\\ L\;(3D) \end{array}\right.\;K_{ms}=\frac{E_{1}}{2(1+v)}\left\{\begin{array}{l} t\;(2D)\\ L\;(3D) \end{array}\right.\\ C_{ks}=\frac{\eta_{2}}{2(1+v)}\left\{\begin{array}{l} t\;(2D)\\ L\;(3D) \end{array}\right.\;K_{ks}=\frac{E_{2}}{2(1+v)}\left\{\begin{array}{l} t\;(2D)\\ L\;(3D) \end{array}\right. \end{array}\right.$$

where $C_{mn}$, $K_{mn}$, $C_{kn}$ and $K_{kn}$ are the four normal parameters of the Burger’s contact model, and $C_{ms}$, $K_{ms}$, $C_{ks}$ and $K_{ks}$ are the four tangential parameters of the Burger’s contact model. The macroscopic Burger’s parameters of the asphalt mortar can be determined through laboratory tests. Since the Burger’s contact between mortar units is composed of two viscoelastic asphalt mortars in series, the relationship between the Burger’s model parameters of the mortar units themselves and the contact Burger’s model parameters is shown in Equation (8).

(8) $$\left\{\begin{array}{l} c_{mn}=2C_{mn}\;,\;k_{mn}=2K_{mn}\\ c_{kn}=2C_{kn}\;,\;k_{kn}=2K_{kn}\\ c_{ms}=2C_{ms}\;,\;k_{ms}=2K_{ms}\\ c_{ks}=2C_{ks}\;,\;k_{ks}=2K_{ks} \end{array}\right.$$

The $\eta_{1}$, $\eta_{2}$, $E_{1}$ and $E_{2}$ four parameters of asphalt mortar need to be determined. Particles and asphalt smaller than 2.36 mm were defined as asphalt mortar. The Poisson’s ratio of asphalt mortar is 0.5 [2,42,43]. Its complex modulus and phase angle were determined using the dynamic shear rheometer test. The values were obtained by fitting the Burger’s model parameters to the principal curves of complex modulus and phase angle. The Burger’s model parameters for the four asphalt mortars are listed in Table 3.

#### 2.2.2. Simulation Process

(1) Numerical model of aggregate particles

Scanning tests of real aggregate morphology were carried out using 3D laser scanning methods, and numerical models of aggregates with morphology features such as aggregate shape and angularity were established based on the aggregate particles obtained from the scanning [44].

**Figure 4:**
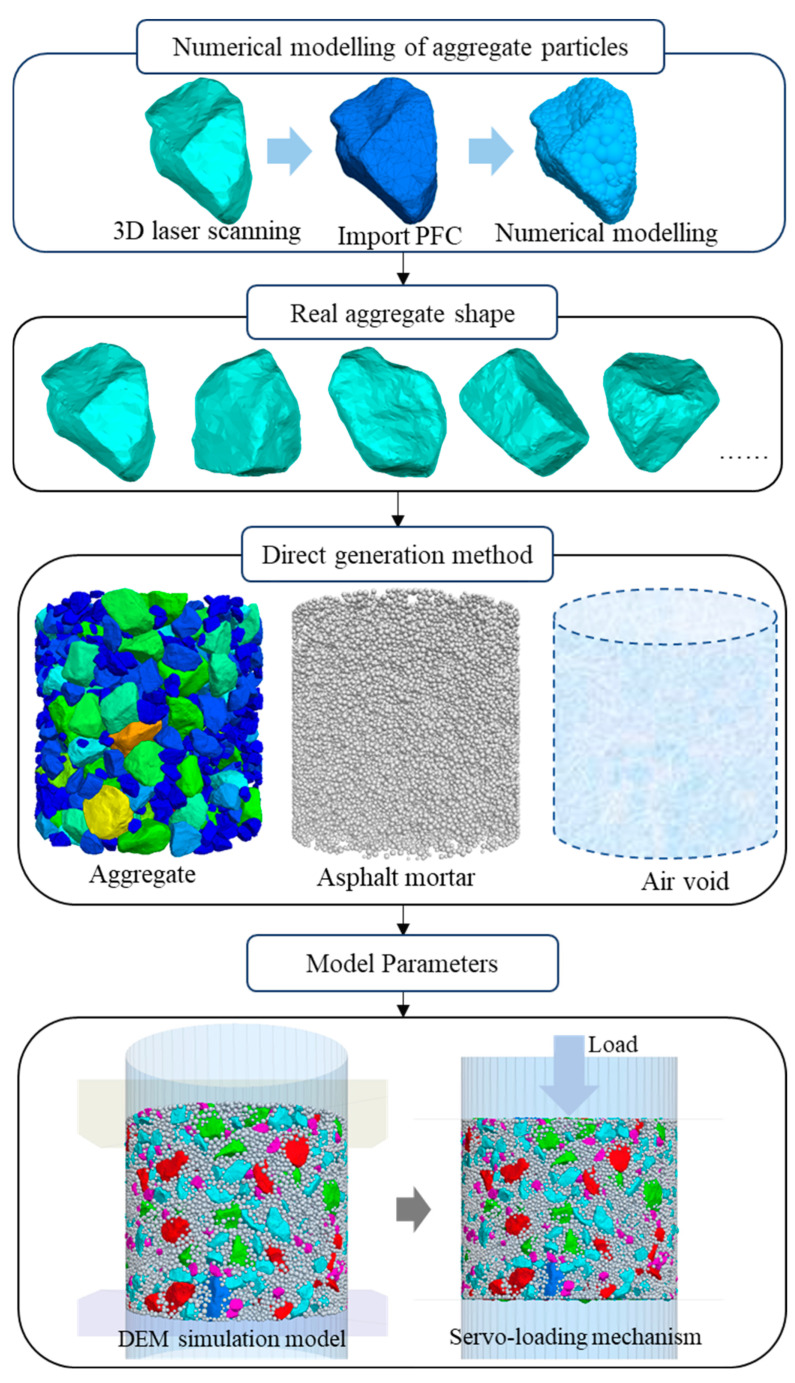
DEM simulation process of asphalt mixture.

According to the 3D aggregate template obtained from scanning, the numerical model of the aggregate is constructed through the fish language in PFC, as shown in Figure 4. The accuracy and efficiency of aggregate particle generation are ensured by setting the parameters related to particle generation. The distance value is set to ensure the smoothness of pebble generation distribution in each clump; the ratio value is set to ensure the range of pebble particle size; and the radfactor parameter is set to control the amount of overlap between the generated pebble and the surface of three-dimensional particles. In this paper, the above three parameters are set to 120, 0.3 and 1.05, respectively. In order to guarantee the efficiency of the simulation calculations and at the same time to ensure the usability of the results, the computational efficiency of the model is taken into consideration in the selection of aggregate particle templates, with five types of aggregate particles being randomly generated through the code written in fish language for each gradation of aggregate particles. The minimum particle size of the generated particles was 1.18 mm.

(2) Distribution of component materials

The numerical model of asphalt mixture is composed of three parts: aggregate, asphalt mortar and voids. The construction process of the model is as follows: firstly, the volume ratio of the components of the mixture is generated, based on the aggregate particles in the asphalt mixture volume fraction; secondly, the required ratio of asphalt mortar is generated, based on the asphalt mixture in the asphalt mortar volume fraction; thirdly, the required proportion of the void is generated, based on the actual void ratio. The numerical model of each component of the asphalt mixture is generated using the above parameters. The generation of aggregate particles with a diameter greater than 2.36 mm was conducted in accordance with the 3D aggregate scanning particle template. Particles smaller than 2.36 mm were defined as asphalt mortar and replaced with spherical particles in the numerical model.

(3) Parameter settings

The generation of a three-dimensional asphalt mixture digital model occurred through the aforementioned steps. The physico-mechanical parameters, such as aggregates, asphalt mortar and voids, and the contact parameters between the components, were inputted for the subsequent study of the load transfer characteristics of the asphalt mixture. The density of each aggregate is shown in Table 2, while the contact model and model parameters are presented in Section 2.2.1. The generation of the discrete element numerical model for asphalt mixtures is illustrated in Figure 4.

(4) Compaction process

The servo control mechanism is employed in the compaction process, with the top wall simulating the load applied by the indenter. The objective of compacting the asphalt mixture is realized by regulating the velocity of the downward movement of the wall, as illustrated in Figure 4. Concurrently, the compaction time is calculated in accordance with the time–temperature equivalence principle [45]. During the compaction process, the stress value of the upper and lower walls is controlled by the servo mechanism to be always 0.6 MPa until the compaction state operation of the asphalt mixture converges to the set system equilibrium state.

#### 2.2.3. Simulation Model Validation

**Figure 5:**
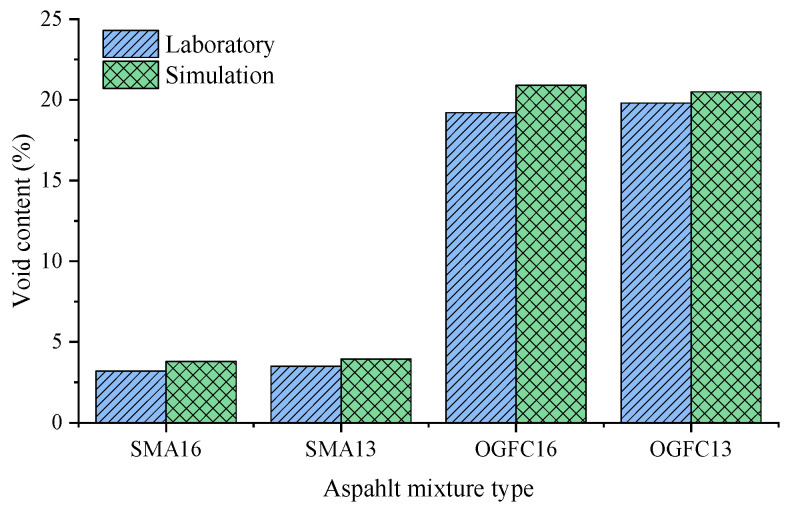
Void content of asphalt mixtures.

As demonstrated in Figure 5, a comparison is presented of the asphalt mixture void ratio obtained using the DEM simulation with the laboratory void ratio. The simulated void ratio has been calculated using the Equation (9).

(9) $$v_{a}=\frac{V_{V}-V_{A}}{V_{V}}\times 100\%$$

where $v_{a}$ is the DEM simulation void ratio of the asphalt mixture; $V_{V}$ is the total volume of the asphalt mixture simulation specimen; and $V_{A}$ is the total volume of aggregate and mortar of the asphalt mixture simulation specimen.

As can be seen from Figure 5, the air void of the simulation test is higher than that of the laboratory tests. In the laboratory test, the aggregate with a smaller particle size and the asphalt can fill the voids between the aggregate particles with a larger particle size. However, due to the computational efficiency of the simulation test, the particle size is constrained to reach the smaller particle size in the laboratory test. Consequently, the void ratio of the asphalt mixtures in the simulation test is larger than that in the laboratory test. The relative error rates between the simulated void ratio and the indoor test void ratio of the four asphalt mixtures (SMA16, SMA13, OGFC16 and OGFC13) were 5.8%, 1.3%, 15.4% and 11.4%, which were all less than 15.4%. The error rate of the simulation in this test was less than that of the value in the study by Gong et al. [38]. The change trend of the void ratio of the four gradations is consistent with that of the laboratory test, and the same trend indicates that the simulation test does not change the overall aggregate skeleton structure. Therefore, DEM simulation can reasonably represent the basic structure of the indoor asphalt mixtures.

## 3. Force Chain Characterization Indicators

### 3.1. Contact Force Distribution Characteristics Indicators

**Figure 6:**
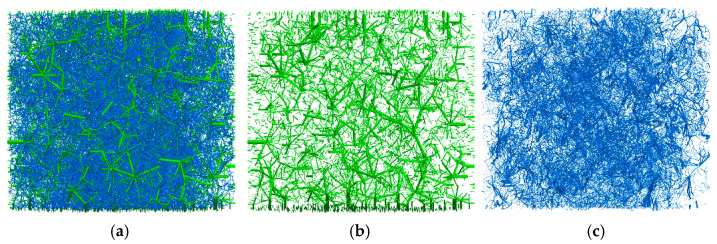
Contact force network of asphalt mixture. (**a**) All contact force; (**b**) Aggregate–aggregate contact force; (**c**) Mortar–mortar/mortar–aggregate contact force. (Note: The green line represents the contact force between aggregate and aggregate particles, while the blue line represents the contact force between mortar and mortar, as well as between mortar and aggregate particles; the force range is 0–159 N; the thicker the line, the larger the force).

Figure 6 shows the contact force network diagram inside the asphalt mixture after compaction of the SMA16 asphalt mixture, in which the thicker the force chain indicates the larger the contact force. The green line represents the contact force between aggregate and aggregate particles, while the blue line represents the contact force between mortar and mortar, as well as between mortar and aggregate particles. As demonstrated in Figure 6, the larger forces are predominantly transferred through the contact network formed by the interaction between aggregate particles, while the smaller forces are primarily transferred between mortar–aggregate and mortar–mortar, which play an auxiliary role in the transfer of loads to the primary skeleton structure. This is consistent with the observation that aggregate particles constitute the principal load-bearing structure of asphalt mixtures.

#### 3.1.1. Average Contact Force

The average contact force for each size aggregate particle is calculated using Equation (10).

(10) $$\bar{F_{s}}=\frac{\sum_{k=1}^{n}f_{k}}{n}$$

where *f_k_* is the magnitude of each contact force value, *n* is the total number of contact forces, and *s* is the particle size.

In order to distinguish the contribution of each component in the load transfer process, the contact forces between aggregate–aggregate and between aggregate–mortar were extracted and calculated separately. The average contact force between aggregate–aggregate is calculated using Equation (11), and the contact force between aggregate–mortar is calculated using Equation (12).

(11) $$\bar{F_{s_{A}}}=\frac{\sum_{k=1}^{n_{A}}f_{k_{A}}}{n_{A}}$$

(12) $$\bar{F_{s_{B}}}=\frac{\sum_{k=1}^{n_{B}}f_{k_{B}}}{n_{B}}$$

where $\bar{F_{s_{A}}}$ and $\bar{F_{s_{B}}}$ denote the average contact force of aggregate–aggregate and aggregate–mortar contacts, respectively; $f_{k_{A}}$ and $f_{k_{B}}$ denote the contact force of aggregate–aggregate and aggregate–mortar contacts, respectively; *n_A_* and *n_B_* denote the number of contacts between aggregate–aggregate and aggregate–mortar, respectively.

#### 3.1.2. Load Bearing Contribution

In order to characterize the contribution of aggregate particles in an asphalt mixture to resist the external load, the ratio of the average contact force of the size *S* particles to the overall average contact force is defined as the load-bearing contribution of size S particles, as shown in Equation (13). This parameter is used to quantify the magnitude of the effect of different aggregate particle sizes in resisting loads. The higher the value, the greater the contribution of that aggregate to the load-bearing capacity of the asphalt mixture.

(13) $$R_{S}=\frac{\bar{F_{s}}}{\bar{F}}$$

where, $R_{s}$ represents the contribution of the *S*th grain size aggregate particles to resist external load; $\bar{F}$ represents the overall average contact force of the asphalt mixture; $\bar{F_{S}}$ represents the average contact force of the *S*th grain size aggregate particles.

The contact force between granular particles can be categorized into two distinct types: aggregate–aggregate contact force and aggregate–mortar contact force. This paper proposes a methodology for evaluating the resistance of these two contact forces to external loads, which involves calculating the ratio of the average value of each contact force type to the average value of the aggregate contact force in the given gradation. The ratio is utilized to ascertain the contribution of each contact type to the overall load-bearing capacity, which can be calculated using Equation (14).

(14) $$\left\{\begin{array}{l} P_{S_{A}}=\frac{\bar{F_{S_{A}}}}{\bar{F_{S}}}\\ P_{S_{B}}=\frac{\bar{F_{S_{B}}}}{\bar{F_{S}}} \end{array}\right.$$

where $P_{S_{A}}$ and $P_{S_{B}}$ are the load-bearing capacity contributions for aggregate–aggregate contact and aggregate–mortar contact, respectively.

#### 3.1.3. Contact Force Angle

**Figure 7:**
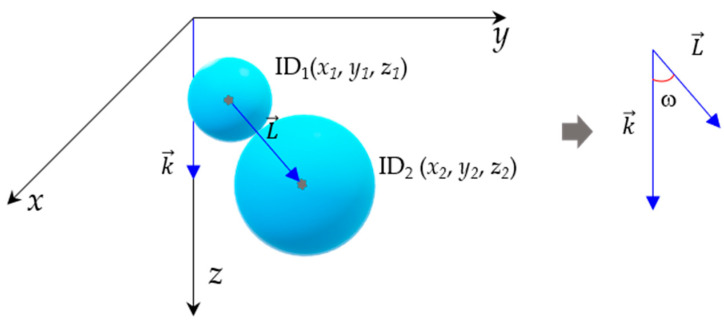
Schematic diagram of contact force angle.

The angle between the position vectors of the particles at the two ends of the contact force and the *z*-axis is defined as the angle of the contact force (ω), as illustrated schematically in Figure 7. It can be simplified to the angle between the vectors formed by the two points and the *z*-axis, which can be calculated using Equation (15).

The larger the angle of contact force, the wider the range of external load transfer that the contact is subjected to; conversely, it indicates that the transfer of load at the contact is concentrated in the loading direction.

(15) $$\vec{k}\cdot \vec{L}=\left|k\right|\left|L\right|\cos\omega$$

(16) $$\cos\omega =\frac{\vec{k}\cdot \vec{L}}{\left|k\right|\left|L\right|}\Rightarrow \omega =\arccos\frac{\vec{k}\cdot \vec{L}}{\left|k\right|\left|L\right|}$$

where $\vec{k}$ is the unit vector in the forward direction of the *z*-axis; $\vec{L}$ is the direction vector between ID1 particles and ID2 particles; and *ω* is the angle of the contact force.

### 3.2. Topology Characterization Indicator of Force Chain

#### 3.2.1. Topology Theory

**Figure 8:**
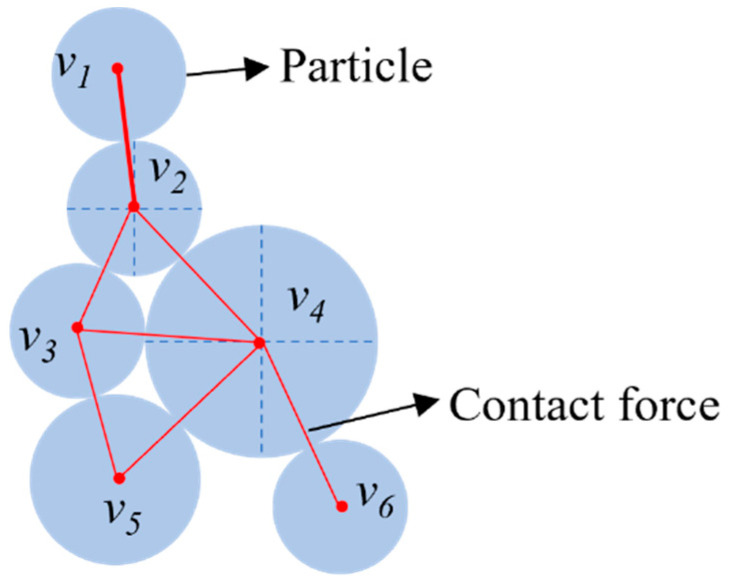
Topology diagram of force chain network.

Force chain architectures in asphalt mixture exhibit topological signatures that can be quantified through topological structure metrics [12,21,22]. Individuals in a topology are considered as nodes of a network. Interactions between individuals are considered as connections between nodes of the network. This network structure can be represented in the form of a graph. A graph can be defined as a triple $G=\left\{V,E,\psi \right\}$, where $E=\left\{e_{1},e_{2},\ldots ,e_{M}\right\}$ is the set of edges and $V=\left\{v_{1},v_{2},\ldots ,v_{N}\right\}$ is the set of nodes. Each connected edge $e_{m}$ in *E* has a pair of nodes $\left(v_{i},v_{j}\right)$ corresponding to it. An association function maps each edge in E to a node in *V*. ψ is defined as a mapping from the set of edges *E* to the set of nodes *V*, and it is termed an association function. Each particle in asphalt mixtures is considered as a node, and the presence of connecting edges between particles is determined based on the presence of a contact force relationship between two aggregates, thus, constituting a network topology between particles in an asphalt mixture [12]. A sketch of the asphalt mixture contact force network is shown in Figure 8.

There are two properties of topological structures: deterministic and stochastic. The determinism of the topological structure is contained in the statistical properties and, thus, it is of great interest to study the structure of complex force chain networks through topological statistics. There are three basic parameters to characterize the network topology: (1) the clustering coefficient that describes the characteristics of the nodes; (2) the edge betweenness reflecting the influence of the edges formed by two nodes in the whole network; and (3) the average path length that reflects the global characteristics of the network [12,23]. According to the analytical characterization needs of this study, the above three topology parameters are selected to characterize the force chain network of the asphalt mixture.

#### 3.2.2. Clustering Coefficient

The clustering coefficient is a statistical value that reflects the degree of aggregation of particle (aggregate or mortar) nodes. It can be used to evaluate the connectivity of the nodes with neighboring nodes and can be calculated using Equation (17).

(17) $$C_{i}=\frac{2e_{i}}{k_{i}(k_{i}-1)},\;C_{i}\in [0,1]$$

where *k_i_* is the degree of particle *i* (node) and *e_i_* is the number of edges between neighboring nodes of node *i*.

The average clustering coefficient (*C*) can be calculated using Equation (18).

(18) $$C=\frac{1}{N}\sum_{i}^{N}C_{i}$$

where *N* is the total number of nodes, i.e., the number of particles forming the skeleton structure.

The greater the clustering coefficient of aggregate particles in the aggregate contact structure of an asphalt mixture, the greater the degree of aggregation of the whole structure and the closer the direct relationship between the aggregate particles. It indicates that the mix has a more optimal bearing structure.

#### 3.2.3. Edge Betweenness

The number of shortest paths between nodes $v_{l}$ and $v_{m}$ is denoted by $N_{lm}$, and the proportion of the number of all shortest paths in the network that pass through an edge $e_{ij}$ is defined as the edge betweenness ($B_{ij}$), which is calculated using Equation (19).

(19) $$B_{ij}=\sum_{\begin{array}{l} Alll,m:l\neq m\\ \left\{l,m\right\}\neq \left\{i,j\right\} \end{array}}\left[N_{lm}(e_{ij})/N_{lm}\right]$$

where $N_{lm}(e_{ij})$ denotes the number of shortest paths between nodes $v_{l}$ and $v_{m}$ through edge $e_{ij}$.

The larger the edge betweenness of the aggregate–aggregate in the force chain in asphalt mixture, the greater the contribution of this edge to resisting the external load.

#### 3.2.4. Average Path Length

The average distances between all pairs of nodes are defined as the average path length (*L*), which can reflect the global characteristics of the force chain network. The average path length can be calculated according to Equation (20).

(20) $$L=\frac{1}{N^{2}}\sum_{j=1}^{N}\sum_{i=1}^{N}d_{ij}$$

where N is the number of nodes; L is the average path length of the force chain network.

The smaller the average path length value in the force chain network of asphalt mixture, the better the connectivity between the aggregates and the more stable the overall structure of the asphalt mixture.

## 4. Results and Discussion

### 4.1. Contact Force Distribution Characteristics in Asphalt Mixtures

#### 4.1.1. Average Contact Force of Particles

(1) Contact force distribution

**Figure 9:**
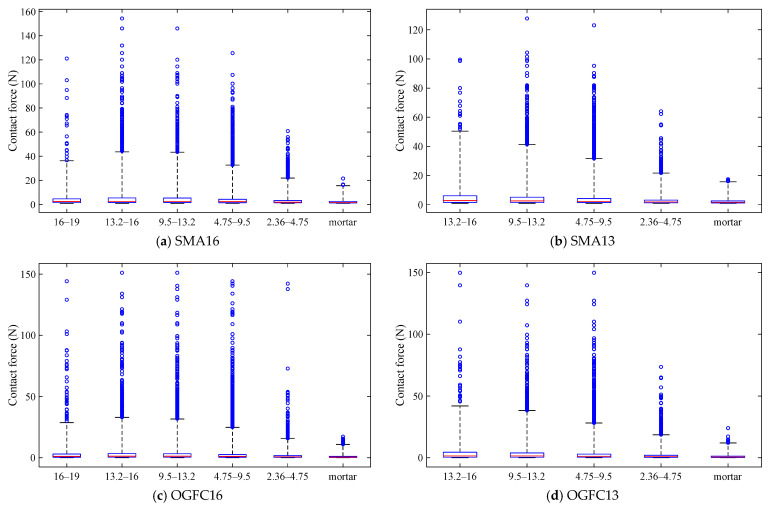
Contact force distribution of each grain size particle in asphalt mixture. (Note: The blue box represents the interquartile range (IQR) spanning from the first quartile (Q1, bottom edge) to the third quartile (Q3, top edge). A bold red horizontal line within the box marks the median value. Blue circles indicate outliers lying beyond the whiskers, highlighting values deviating significantly from the central tendency of the data. The meaning of the box plot below is the same as this and will not be repeated).

The boxplot of the contact force distribution of each grain size aggregate particles in the asphalt mixture is shown in Figure 9. As can be seen from Figure 9, the distribution of contact force anomalies for each size aggregate is concentrated on the side of the larger value, and the contact force distribution shows a right-skewed state. With the decrease of aggregate particle size, the trend of contact force distribution range gradually decreases. The contact force range of the mortar is the smallest, and the maximum value of the distribution range is the smallest. It indicates that in asphalt mixtures, aggregate particles bear and transfer larger external loads. Meanwhile, the role of aggregate particles in constituting the load-bearing skeleton structure was verified using the meso-scale.

(2) Maximum contact force and average contact force

**Figure 10:**
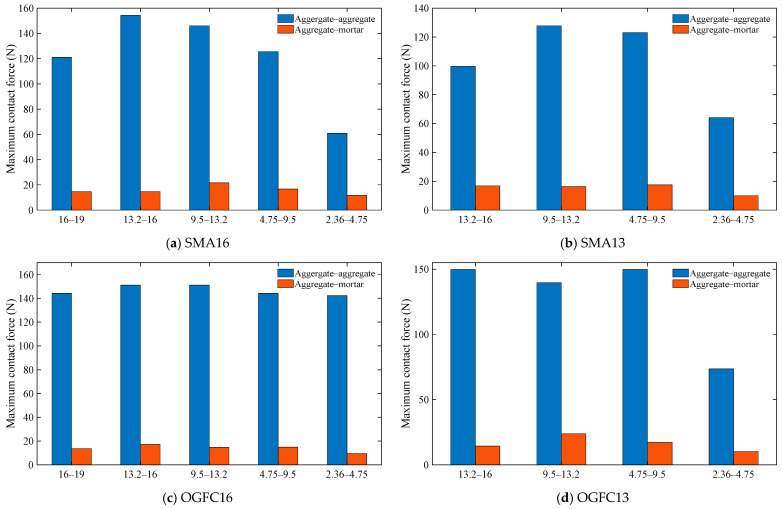
Maximum contact force for different contact types of aggregate particles.

Figure 10 depicts the maximum contact force values for different contact types in the aggregate particles of the asphalt mixture. From Figure 10, it can be seen that the maximum contact force for aggregate–aggregate contact is greater than that for aggregate–mortar contact. For SMA16 and SMA13 asphalt mixtures, the maximum aggregate–aggregate contact force shows a tendency to increase and then decrease with decreasing particle size. For the OGFC16 asphalt mixture, the variation of aggregate–aggregate contact force with different-sized aggregates is smaller. For the OGFC13 asphalt mixture, the variation of aggregate–aggregate contact force with particle size is smaller when the particle size is larger than 4.75 mm, but the average contact force of aggregate particles with particle sizes of 2.36 mm becomes significantly smaller. The above results indicate that the magnitude of inter-particle contact force is related to the differences in the gradation of asphalt mixtures.

**Figure 11:**
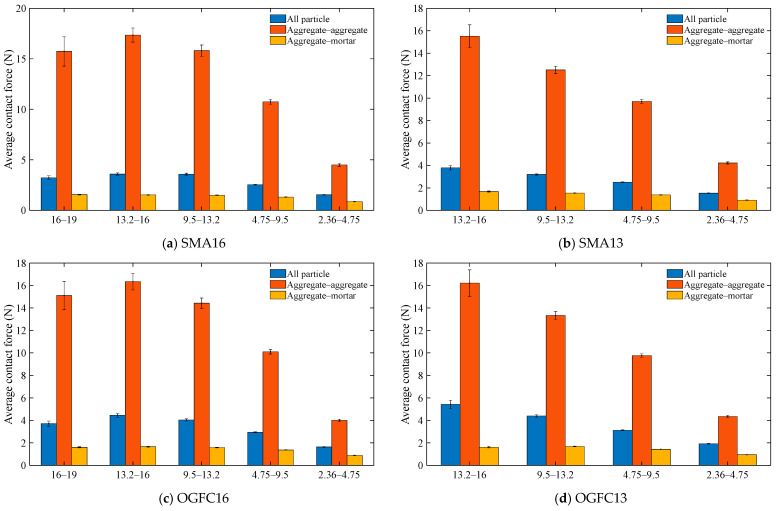
Aggregate contact force for different contact types.

The average contact force for each size of aggregate particles after compaction and the average contact force between aggregate–aggregate particles and between aggregate–mortar in that size of aggregate particles are quantified. Figure 11 shows the average contact force for different contact types in different aggregate particle sizes. It can be seen from Figure 11 that the average contact force gradually decreases as the aggregate particle size decreases. For each size of aggregate particles, the average contact force of aggregate–aggregate contact is significantly greater than the average contact force of the particles, and the average contact force of the aggregate–mortar contact is the smallest. This is mainly due to the fact that the modulus of the asphalt mortar is much smaller than the modulus of the aggregate particles, and in asphalt mixtures under external loading, the aggregate–aggregate contact transmits a greater external load, and the aggregate–mortar contact and the mortar–mortar contact transmit a smaller load.

#### 4.1.2. Load-Bearing Contribution of Particles

**Figure 12:**
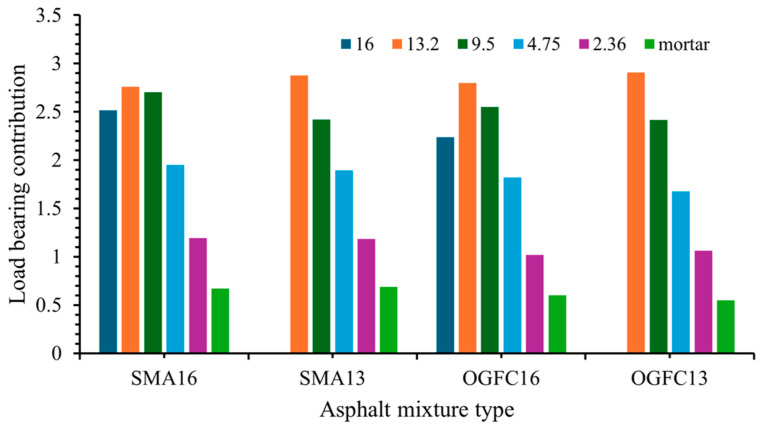
Load-bearing contribution of different-sized particles.

The load-bearing contribution of different-sized particles is shown in Figure 12. It can be seen from Figure 12 that for the SMA16 and OGFC16 asphalt mixtures, the load-bearing contribution shows an increase and then a decrease as the particle size decreases. When the aggregate particle size is greater than 13.2 mm, the load-bearing contribution decreases with increasing particle size. When the aggregate particle size is less than 13.2 mm, the load-bearing contribution decreases with decreasing particle size. For the SMA13 and OGFC13 asphalt mixtures, the aggregate particles’ load-bearing contribution decreases with decreasing particle size. It indicates that when the nominal maximum particle size of the asphalt mixture is less than 13.2 mm, the load-bearing contribution of aggregate increases with the increase of particle size. The load-bearing contribution of mortar in all four asphalt mixtures is less than one, which indicates that mortar is not the main participant in the bearing structure of the asphalt mixtures. For the same size aggregate particles, the value of the load-bearing contribution is obviously different in different asphalt mixtures. The above results indicate that the load-bearing contribution of different-sized aggregates to the overall structure is different, and the load-bearing contribution of the same-sized aggregate is affected by the gradation type of the asphalt mixture. Related research results also confirm that the load-bearing contribution is more significantly affected by gradation [46]. Load-bearing contribution is higher in large-size aggregates above 9.5 mm, which exhibit greater load transfer efficiency [10].

**Figure 13:**
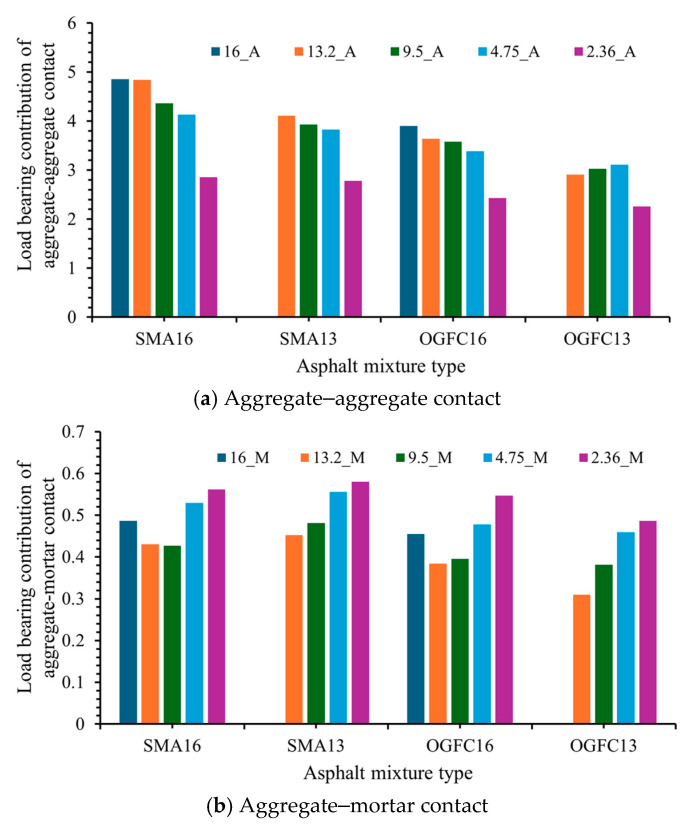
Load-bearing contribution of different contact types.

The load-bearing contributions of different contact types of aggregates in asphalt mixtures are shown in Figure 13. From Figure 13a,b, it can be seen that the load-bearing contribution of aggregate–aggregate contact ($P_{S_{A}}$) is significantly larger than that of aggregate–mortar contact ($P_{S_{B}}$). For the four asphalt mixtures, the aggregate–aggregate contact type shows a gradual decrease in load-bearing contribution with decreasing aggregate particle size. This may be due to the fact that the modulus of the large-size aggregate particles changed little even after adhering to the asphalt mortar, and the bearing capacity did not change much due to the adhesion of the mortar, whereas the modulus of the smaller-sized aggregate particles was affected more by the mortar after adhering to the asphalt mortar, which in turn affected the bearing capacity, so the value decreased gradually with the decrease in the aggregate particle size. This phenomenon illustrates why the value gradually increases with decreasing aggregate particle size in different gradations. As demonstrated in Figure 13, for a given particle size in different asphalt mixtures, the load-bearing contribution value of aggregate–aggregate contact for SMA16 is the largest, followed by SMA13, and then OGFC16, with OGFC13 being the smallest. This also reflects that the SMA has a superior load-bearing contact structure. It resists deformation under repeated loads, which is a key factor in rutting resistance [23].

#### 4.1.3. Contact Force Angle of Particles

**Figure 14:**
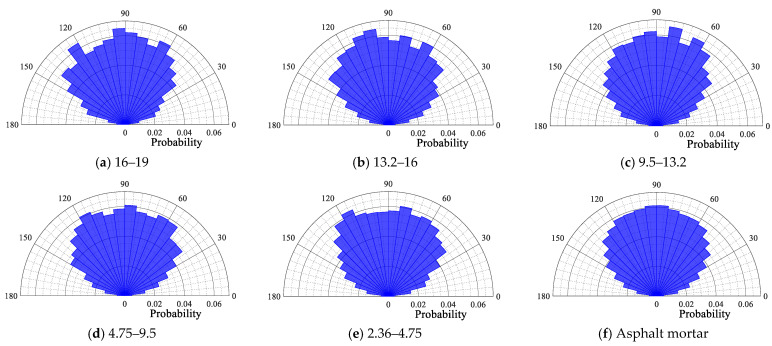
Contact force angle for each particle size in SMA16.

**Figure 15:**
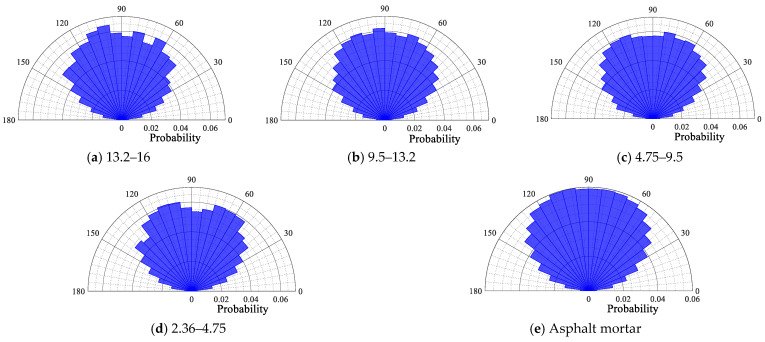
Contact force angle for each particle size in SMA13.

**Figure 16:**
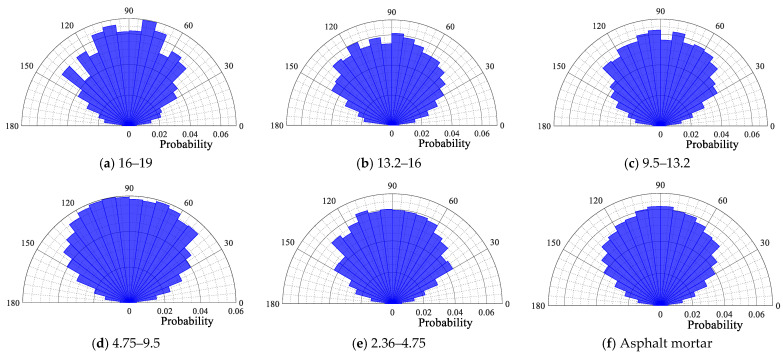
Contact force angle for each particle size in OGFC16.

**Figure 17:**
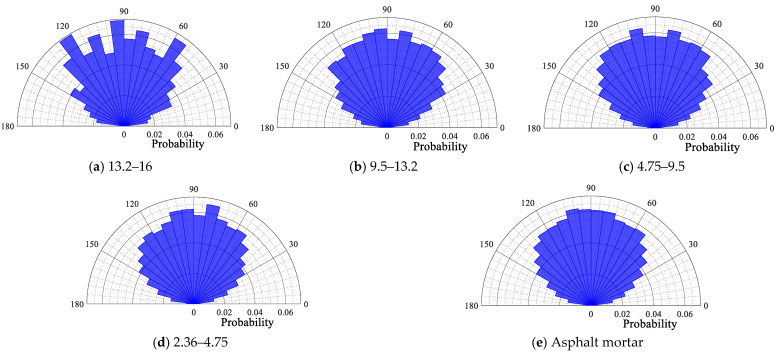
Contact force angle for each particle size in OGFC13.

The probability distribution polar plot of the contact force angle of each size particle is depicted in Figure 14, Figure 15, Figure 16 and Figure 17. From Figure 14, Figure 15, Figure 16 and Figure 17, it can be seen that for the four gradations, the contact force angle of each particle size is symmetrically distributed around a center axis of 90°, with the contact force angle centrally distributed within the range of 30° to 150°. The results demonstrate that the external load is predominantly transmitted along the loading direction to the external extension in the internal transmission process of the asphalt mixture. This outcome is consistent with the results of the aforementioned loading process in the asphalt mixture, wherein the contact force is transmitted along the direction of the external load.

### 4.2. Force Chain Topology Characteristics in Asphalt Mixtures

#### 4.2.1. Clustering Coefficient of Asphalt Mixture

**Figure 18:**
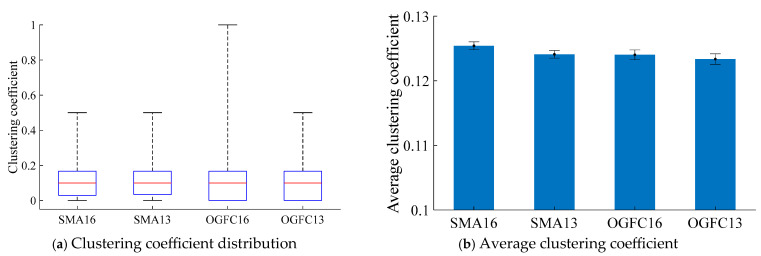
Clustering coefficient of different asphalt mixtures.

The clustering coefficients of different asphalt mixtures are presented in Figure 18. The distribution of clustering coefficients is given in Figure 18a, and it can be seen that the distribution of clustering coefficients at all asphalt mixtures demonstrates a right-skewed distribution, with outliers located on the side of the larger quartile. This indicates that in the asphalt mixture bearing skeleton structure, the number of particles that play a major bearing role accounts for a relatively small proportion. This is primarily due to the generated asphalt mixture DEM model, the asphalt mortar particles particle size being small and, thus, the number of particles being relatively large. From the above analysis, it can be seen that the asphalt mortar is not a major bearing structure. Therefore, the distribution of clustering coefficients values appears to have a severely skewed distribution phenomenon.

The average clustering coefficients are shown in Figure 18b. As illustrated in Figure 18b, the average clustering coefficient of SMA13 is lower than that of SMA16 by 0.0013. This finding suggests that the aggregate particles of SMA16 exhibit enhanced connectivity, characterized by a higher degree of aggregation compared to SMA13. Consequently, it indicates that the load-bearing skeleton structure of SMA16 is superior to that of SMA13. The average clustering coefficient of the OGFC16 mixture is greater than that of the OGFC13 by 0.0007, indicating that the load-bearing structure of the OGFC16 mixture is superior to that of the OGFC13. A comparison of the different gradation asphalt mixtures, with the same maximum nominal particle size, reveals that the average clustering coefficients of the SMA asphalt mixtures exceed those of the corresponding OGFC asphalt mixtures. The average clustering coefficient of SMA16 is greater than OGFC16 by 0.0014. This finding demonstrates that the OGFC asphalt mixtures possess greater load-bearing requirements for aggregate particles and that the SMA-type gradations demonstrate a more effective aggregate load-bearing structure than the OGFC-types.

#### 4.2.2. Edge Betweenness of Asphalt Mixture

**Figure 19:**
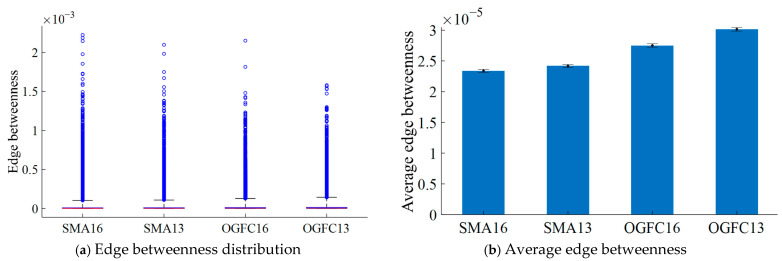
Edge betweenness of different asphalt mixtures.

The edge betweenness distribution and the average edge betweenness of different asphalt mixtures are shown in Figure 19. As can be seen from Figure 19a, the distributions of the edge betweenness of different asphalt mixtures all show right skewed distributions, and the outliers are located on the side of the larger quartiles. This is primarily attributable to the fact that, within the generated DEM model of the asphalt mixture, the number of particle pairs (edges formed by two particles) that play a major role in load-bearing structure is comparatively minimal. The particle size of asphalt mortar particles is small and, thus, the number of mortar particles is relatively large, but the edge formed between the mortar particles does not play a major role in the load-bearing structure and, thus, smaller values of edge betweenness account for more.

As can be seen from Figure 19b, the average edge betweenness of SMA16 is smaller than that of SMA13 by 7.95 × 10^−7^. Additionally, the average edge betweenness in OGFC16 is smaller than that in OGFC13 by 2.65 × 10^−6^. It also can be seen that the average edge betweenness in SMA-type asphalt mixture is smaller than that in OGFC-type asphalt mixture with the same maximum nominal particle size. OGFC16 improves by 4.12 × 10^−6^ relative to SMA16 and OGFC13 improves by 5.98 × 10^−6^ relative to SMA13. The results presented above demonstrate that the edge between two particles in SMA13 contributes more to the force chain network structure than SMA16; the edge between two particles in OGFC13 contributes more to the force chain network structure than OGFC16. Additionally, the edge between particles in SMA-type mixtures contributes less to the overall contact force structure than OGFC-type mixtures.

#### 4.2.3. Average Path Length of Asphalt Mixture

**Figure 20:**
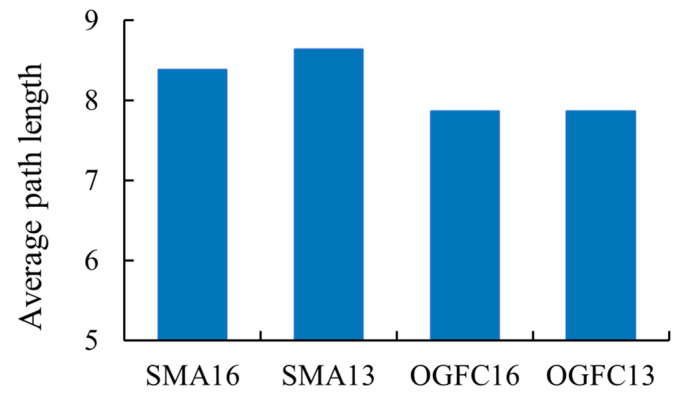
Average path length of different asphalt mixtures.

**Figure 21:**
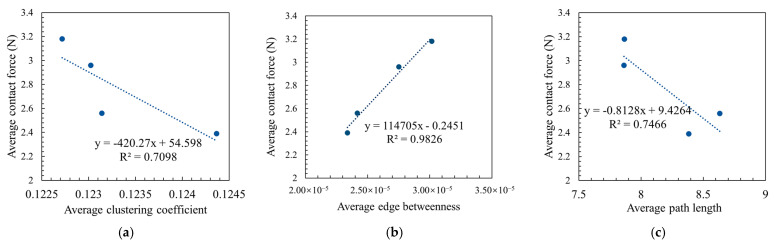
Correlation of the average contact force with the topological characteristics of the force chain. (**a**) Average contact force and average clustering coefficient. (**b**) Average contact force and average edge betweenness. (**c**) Average contact force and average path length.

The average path length of the force chain structure in asphalt mixtures is depicted in Figure 20. As illustrated in Figure 20, the average path length of SMA13 is marginally greater than that of SMA16, exhibiting a difference of 0.25. Similarly, the average path length of OGFC13 is slightly higher than that of OGFC16, with a difference of 0.003. Furthermore, the average path length of the SMA-type mixtures exceeds that of the OGFC-type mixtures. The relative change rate in the average path length between the same type of gradation with different maximum nominal grain sizes is less than 3%. The relative change rate in the average path length between different types of gradations (OGFC and SMA) is 7% to 9%. This finding indicates that the maximum nominal particle size exhibits a lower influence on the average path length, while the type of gradation exhibits a more significant effect. According to the concept of average path length in topology and the above analyses, it can be found that asphalt mixtures of the same gradation type have similar load-bearing structures, even though they have different maximum nominal particle sizes. SMA asphalt mixtures have more stable load-bearing structures than OGFC asphalt mixtures. This finding is consistent with the results of the characterization of the contact force distribution of asphalt mixtures in Section 4.1. Based on the analyses in Section 4.1 and Section 4.2, it can be seen that the key influential parameters for the load transfer characterization of asphalt mixtures are shown in Table 4. For all four asphalt mixtures, the average contact force and topological structure parameters better reflect the load transfer characteristics within the asphalt mixtures. Additionally, the correlation between the contact force distribution characteristics and the force chain topology characteristics is shown in Figure 21, where the coefficients of determination (*R*^2^) are all greater than 0.7. This indicates that there is a strong correlation between the average contact force and the force chain topology parameters. The related study also found that there is a correlation between statistical and topological features of the force chain [12,23].

## 5. Conclusions

In this study, a series of DEM simulation tests were performed considering SMA gradations and OGFC gradations to characterize the contact force distribution in the asphalt mixtures. Additionally, the force chain topology characterization was also analyzed based on topology theory. The primary conclusions are as follows.

(1) The contact forces of aggregate particles of varying grain sizes in asphalt mixtures are distinct. The average contact force of particles in SMA16 and OGFC16 exhibits a tendency to increase and subsequently decrease with decreasing particle size, with the maximum average contact force recorded for a particle size of 13.2 mm. For SMA13 and OGFC13 asphalt mixtures, the average contact force demonstrates a gradual decline with decreasing particle size.
(2) The particles with different sizes in the asphalt mixture have different load-bearing contributions. For SMA16 and OGFC16 asphalt mixtures, the load-bearing contribution initially increases and then decreases with decreasing aggregate size, reaching a maximum at 13.2 mm. For SMA13 and OGFC13 asphalt mixtures, the aggregate particles’ load-bearing contribution decreases with decreasing particle size. The load-bearing contributions of different contact types in asphalt mixtures are different. The $P_{S_{A}}$ is significantly larger than $P_{S_{B}}$. The $P_{S_{A}}$ for SMA16 is the largest, followed by SMA13, and then OGFC16, with OGFC13 being the smallest.
(3) The contact force angle distribution characteristics are less affected by the type of asphalt mixture. The contact force angle of each particle size is symmetrically distributed around a center axis of 90°, with the contact force angle centrally distributed within the range of 30° to 150°.
(4) The topological characteristic parameters can effectively characterize the structural features of the asphalt mixture’s force chain network. The average clustering coefficients of SMA are larger than those of the OGFC. The SMA asphalt mixture has a better load-bearing force chain structure than the OGFC asphalt mixture. The average clustering coefficient of SMA13 is smaller than that of SMA16. The load-bearing force chain structure of SMA16 is better than that of SMA13. The average clustering coefficient of OGFC16 is larger than that of OGFC13. The force chain structure of OGFC16 is better than that of OGFC13.
(5) The edge betweenness of the aggregate–aggregate in the force chain in SMA asphalt mixture is smaller than that in OGFC asphalt mixture. Additionally, the average edge betweenness in SMA16 is smaller than that in SMA13. The edge between two particles in SMA13 contributes more to the force chain network structure than SMA16; the edge between two particles in OGFC13 contributes more to the force chain network structure than OGFC16.
(6) The gradation type of asphalt mixture has a significant effect on the average path length of the force chain network, and the maximum nominal particle size of the same gradation has a lesser effect on the average path length. The relative change rate in average path length for the same gradation type of asphalt mixture with different maximum nominal particle sizes is 3%, while the relative change rate in the average path length of the force chain network for different gradation types of asphalt mixtures is 7% to 9%.

The topological parameters proposed in this paper can be used to guide the grading optimization. According to the above results, in comparison to OGFC, SMA has a larger average clustering coefficient and denser force chain network, and the aggregate particles are well connected to each other, forming a more effective load-bearing skeleton structure. This structure enables SMA mixtures to better resist deformation and reduce rutting when subjected to vehicle loads. SMAs have a better topology and load-bearing skeletal structure compared to OGFC mixtures, and their compact skeletal structure also contributes to resisting volume changes during freeze–thaw cycles and improving frost resistance. In future studies, the grading of OGFC can be optimized to improve aggregate interlocking in OGFC, and to improve mix durability without compromising drainage. Gradation optimization can be performed using force chain topological network analysis. However, it is recommended that the results be validated by further laboratory testing to refine the understanding of the relationship between force chain characteristics and mechanical properties of asphalt mixtures.

## Figures and Tables

**Table 1:** Selected gradations.

Sieve Size (mm)	Percent Passing (%)			
	SMA13	SMA16	OGFC13	OGFC16
19	-	100	-	100
16	100	95.8	100	96.4
13.2	93.2	74.7	94.9	79.3
9.5	61.3	55.3	70.5	57.9
4.75	25.8	25.9	22.1	22.9
2.36	19.9	20.4	14.4	16.0
1.18	16.0	17.2	9.8	11.2
0.6	13.7	14.6	7.2	8.4
0.3	12.2	13.1	5.4	6.6
0.15	10.6	11.4	4.4	5.4
0.075	9.8	10.4	3.8	4.7
Optimal asphalt–aggregate ratio	6.0%	5.9%	4.4%	5.2%

**Table 2:** Bulk density of aggregates.

Aggregates	Size (mm)	Bulk Density (g/cm^3^)
Coarse aggregates	16–19	2.892
	13.2–16	2.829
	9.5–13.2	2.764
	4.75–9.5	2.762
	2.36–4.75	2.751
Fine aggregates	1.18–2.36	2.700
	0.6–1.18	2.682
	0.3–0.6	2.602
	0.15–0.3	2.751
	0.075–0.15	2.651
Mineral Powder	<0.075	2.806

**Table 3:** Burger’s model parameters of four asphalt mortars.

Asphalt Mixtures	*E_1_* (MPa)	*E_2_* (MPa)	$\eta_{1}$ (MPa·s)	$\eta_{2}$ (MPa·s)
SMA16	183.4	10.5	161.3	20.3
SMA13	79.6	3.7	18.2	6.8
OGFC16	153.9	6.8	102.0	20.3
OGFC13	223.0	20.1	101.2	1.63

**Table 4:** Key parameters of force chain characteristics for asphalt mixtures.

Asphalt Mixture	Key Parameters	
	Distribution Characteristics	Topology Characteristics
SMA16	Average contact force ($\bar{F_{s}}$)	Average clustering coefficient (*C*) Average edge betweenness ($\bar{B_{ij}}$) Average path length (*L*)
SMA13		
OGFC16		
OGFC13		

## Data Availability

The original contributions presented in this study are included in the article. Further inquiries can be directed to the corresponding authors.
